# Circulating gamma‐glutamyl transpeptidase and risk of pancreatic cancer: A prospective cohort study in the UK Biobank

**DOI:** 10.1002/cam4.5556

**Published:** 2022-12-29

**Authors:** Weiting Liao, Yu Yang, Huazhen Yang, Yuanyuan Qu, Huan Song, Qiu Li

**Affiliations:** ^1^ Department of Medical Oncology Cancer Center, West China Hospital, Sichuan University Chengdu China; ^2^ West China Biomedical Big Data Center Sichuan University Chengdu China; ^3^ Medical Big Data Center Sichuan University Chengdu China

**Keywords:** European ancestry, gamma‐glutamyl transpeptidase, pancreatic cancer, prevention

## Abstract

**Background:**

To determine whether serum gamma‐glutamyl transpeptidase (GGT) level is associated with pancreatic cancer risk in a large prospective cohort.

**Methods:**

The study analyzed serum GGT concentration at baseline of 421,032 participants recruited in the UK Biobank since 2006 through 2010. Information on incidence of pancreatic cancer was obtained from cancer and death registers, updated until 2015 in Scotland or 2016 in England and Wales. Adjusted Cox proportional hazards models were used to measure the association between serum GGT and pancreatic cancer risk.

**Results:**

The study identified 586 cases of pancreatic cancer over a median follow‐up period of 7.16 years. In the multivariable‐adjusted Cox model, serum GGT level was associated with 14% higher pancreatic cancer risk (hazard ratio (HR) per one standard deviation increment of log2 GGT level = 1.14, 95% confidence interval (CI) 1.02–1.28, *p* = 0.025). In the total population, the HR for the highest GGT group was 1.68 (95%CI: 1.22–2.30) versus the lowest GGT group. The HR for the highest GGT group in men (≥50.2 U/L) was 1.72 (95%CI: 1.14–2.61) and that in women (≥31.6 U/L) was 1.75 (95%CI: 1.06–2.88) versus the lowest GGT group.

**Conclusion:**

Our findings suggested a positive association of serum GGT in pancreatic cancer etiology, implying the potential of monitoring GGT level for identifying at‐risk individuals for pancreatic cancer.

## Graphical abstract

The study analyzed serum GGT concentration at baseline of 421,032 participants recruited in the UK Biobank since 2006 through 2010. Elevated serum GGT level predicted a higher risk of pancreatic cancer after fully adjusting for multiple confoundings. Therefore, serum biomarker GGT may have utility to early detect the high‐risk population with pancreatic cancer.

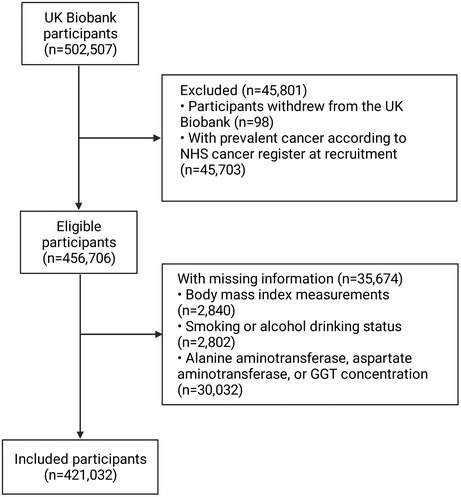

## 1 INTRODUCTION

Pancreatic cancer has the worst prognosis among the common solid tumors, with the five‐year overall survival rate of around 10%.^1^ Because of lack of effective early detection strategies, most patients are diagnosed with pancreatic cancer at late stages.^2^ Many unknowns in pancreatic cancer etiology exist even regarding several well‐known risk factors of this disease including smoking, history of diabetes mellitus, and adiposity.^3^, ^4^

Gamma‐glutamyl transpeptidase (GGT) is an ectoenzyme on the apical plasma membrane of epithelial cells,^5^ widely distributed in human tissues, which could protect against oxidants by replenishing intracellular glutathione.^6^ It is believed that serum GGT is primarily derived from the liver, as a reliable indicator of liver damage caused by alcohol.^7^ Elevated GGT could predict various chronic diseases, such as cardiovascular events, hypertension, type II diabetes, metabolic syndrome, and renal failure.^8^ Discordant results have been reported on the relationship between elevated serum GGT level and cancer incidence of digestive organs, respiratory system, urinary and genital organs, etc.^9^ The existing evidence between serum GGT and incident pancreatic cancer is limited and equivocal.^10^, ^11^, ^12^, ^13^

A complex and multifactorial etiology of pancreatic cancer poses a challenge to identify predisposing risk factors.^14^ The UK Biobank, an ongoing prospective cohort of UK residents, has gathered plenty information on sociodemographic, lifestyle, and biomarker factors as well as health outcomes. Based on this nationwide study, we aimed to examine the impact of sex‐specific GGT level on pancreatic cancer risk.

## 2 MATERIALS AND METHODS

### 2.1 Study participants

The UK Biobank consists of 502,507 adults aged 40–69 enrolled between 2006 and 2010.^15^ Participants were all registered with the UK National Health Service (NHS) with 94% of self‐reported European ancestry. Briefly, besides enriched information collected at baseline by questionnaires on socio‐demographic, lifestyle and health‐related items, participants were required to complete a range of anthropometric measures and offer biological samples.^16^ Cancer incidence and mortality data were followed up by linking to cancer and death records through the NHS Digital for England and Wales and NHS Central Register, National Records of Scotland for Scotland. Complete follow‐up was available until 31 March 2016 for England and Wales and 31 October 2015 for Scotland.^17^

### 2.2 Exclusion criteria

**FIGURE 1:**
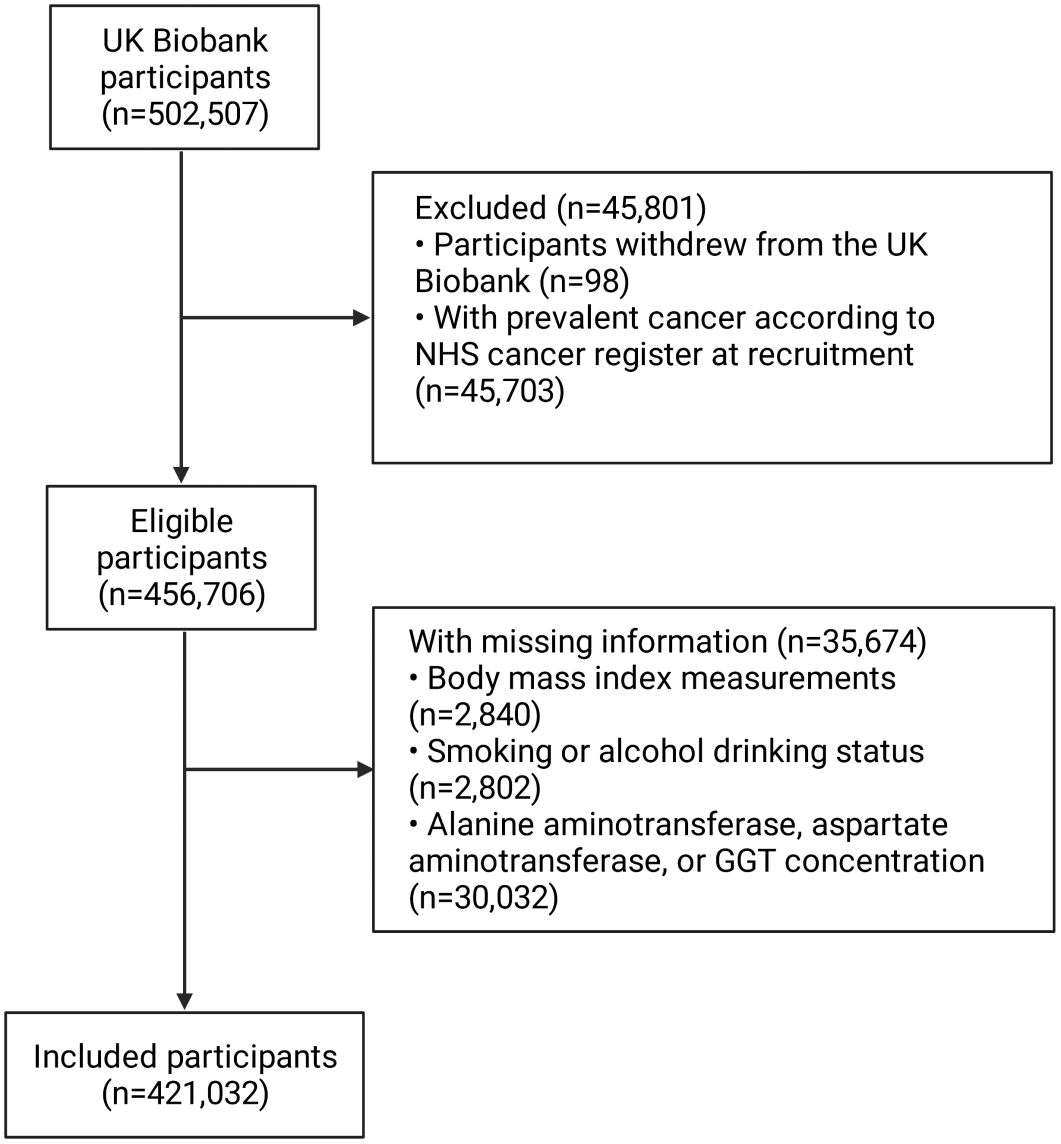
The flow diagram for exclusion and inclusion. We excluded participants who withdrew from the UK Biobank and had prevalent cancers, with missing information on key measurements, leaving 421,032 participants in our study.

Criteria for exclusions were participants withdrew from the UK Biobank (*n* = 98), with prevalent cancer according to NHS cancer register at recruitment (*n* = 45,703), with missing information on body mass index (BMI) measurements (*n* = 2,840), smoking or alcohol drinking status (*n* = 2,802), or results of blood tests for alanine aminotransferase, aspartate aminotransferase, or GGT concentration (*n* = 30,032). Finally, our phenotypic analysis included 421,032 participants, which were then followed up until the date of cancer diagnosis, death, or the last follow‐up, whichever came first. (Figure 1).

### 2.3 Assessment of exposure

As part of the UK Biobank Biomarker Project, serum GGT level was examined by enzymatic rate approach on a Beckman Coulter AU5800 analyzer to the manufacturers' specifications. The detection threshold for GGT level ranged from 5 to 1200 U/L. Sample quality control for GGT assays was conducted by third party Internal Quality Control material from Randox Laboratories and Technopath and externally verified in WEQAS Mainline Chemistry. More information on assay performance have been published previously.^18^

### 2.4 Assessment of outcome

We defined pancreatic cancer cases as the ones with a diagnosis of pancreatic cancer or dying from it during follow‐up based on the Tenth Revision of the International Classification of Diseases (ICD‐10) (i.e., C25) by linking records to the national cancer and death registers.

### 2.5 Assessment of covariates

Self‐completed questionnaires were used to collect sociodemographic data (birth date, gender) and characteristics related to smoking, alcohol consumption, and physical activity. Participants were assigned a Townsend deprivation index that represents their socioeconomic status according to their postcode. The assessment of physical activity was conducted using the International Physical Activity Questionnaire, which was summed over walking, moderate, and vigorous activity as metabolic equivalents (MET min/week). Trained nurses collected data on height, body weight, waist circumference during the initial assessment center visit. All participants were given a touchscreen food frequency questionnaire at recruitment about diet information.

### 2.6 Statistical analysis

The hazard ratios (HRs) and 95% confidence intervals (CIs) of serum GGT level for incident pancreatic cancer were assessed by Cox proportional hazards regression models. GGT was modeled on a continuous scale and also classified into four circulating levels based on sex‐specific quartiles. Log2 transformation of the nonnormally distributed continuous GGT scale was applied to reduce skewness. Z score for GGT standardization was analyzed for a standard deviation (SD) increase in the HR. A series of baseline characteristics were taken into account when fitting the multivariable model (model 1), namely gender, age at recruitment, UK Biobank Assessment Centre, Townsend deprivation index at recruitment, qualifications. In model 2, we additionally adjusted BMI, waist circumference, total physical activity per week, smoking status, alcohol consumption status, and levels of glycated hemoglobin (HbA1c). Furthermore, we adjusted for markers of inflammation and that possibly interrelate/cross talk with the GGT pathway including C‐reactive protein, total bilirubin, alkaline phosphatase, alanine aminotransferase, aspartate aminotransferase, cholesterol, triglycerides, and low‐density lipoprotein direct in model 3. P for trend was also calculated using the median value of each category of GGT concentration as a continuous variable in the models.^19^ Stratified analyses were conducted by age at baseline (<60, ≥60 years), smoking status (never, former or current smokers), alcohol consumption (never, former or current drinkers), BMI category (<25, ≥25 kg/m2) and new‐onset diabetes before the cancer diagnosis within three years (positive or negative) during the follow‐up period. A cross‐product term was included in the multivariate model to examine whether sex‐specific quartile of GGT level interacted with potential risk factors. The likelihood ratio test statistic in regression models with and without multiplicative interaction terms between sex‐specific quartile of GGT level (categorical) and stratified variables (categorical).

Furthermore, we examined the possibility of nonlinearity of log2 transformation of serum GGT level as continuous variables to predict pancreatic cancer using cubic spline models.

We conducted sensitivity analyses to assess the impact of reverse causality after exclusions of the first year of follow‐up, participants with new‐onset diabetes before the cancer diagnosis within three years, inflammatory bowel disease (IBD), liver or bile duct disease and liver‐toxicity medication use (including paracetamol, aspirin, ibuprofen, simvastatin, carbamazepine). Furthermore, we corrected the additional confounding by controlling for dietary lifestyle characteristics, such as raw and cooked vegetable intake, fresh and dried fruit intake, frequency of poultry consumption, and first‐degree family history of digestive cancer.

Statistical tests were all two‐sided and a value of *p* < 0.05 was considered statistically significant. Analyses were conducted using R 3.6.3.

## 3 RESULTS

### 3.1 Study participants

A total of 421,032 participants (196,974 [46.78%] men and 224,058 [53.22%] women) were included in the study. Among the participants, the mean age was 56.23 (SD 8.10) years. In Table 1, the participants' baseline characteristics are listed. Compared with those in the bottom quartile (quartile 1, Q1 for women <16 U/L and for men <23.7 U/L), participants in the top circulating GGT quartile (quartile 4, Q4 for women ≥31.6 U/L and for men ≥50.2 U/L) were elder, less physically active, less likely to have a university degree, had a higher BMI, and were more likely to be current smokers and drinkers. Additionally, participants in the top circulating GGT quartile had higher circulating concentrations of glycated hemoglobin, C‐reactive protein, aspartate aminotransdrease, alanine amnotransferase, alkaline phosphatase, triglycerides, cholesterol, and low‐density lipoprotein direct but lower circulating concentrations of total bilirubin.

**TABLE 1:** Characteristics of UK Biobank participants by category of serum gamma‐glutamyl transpeptidase level. (*n* = 421,032 participants)

Characteristics	Q1	Q2	Q3	Q4
Gamma‐glutamyl transpeptidase concentration, U/L	15.96 (3.82)	22.96 (5.28)	32.49 (8.37)	77.42 (66.63)
*n*	104,596	105,438	105,520	105,478
Pancreatic cancer, *n* (%)	93 (0.09)	143 (0.14)	166 (0.16)	184 (0.17)
Age at base, years	54.72 (8.42)	56.35 (8.14)	56.90 (7.92)	56.94 (7.71)
Sex, female, *n* (%)	55,558 (53.12)	56,177 (53.28)	56,205 (53.26)	56,118 (53.20)
Body mass index, kg/m^2^	25.54 (3.80)	26.85 (4.36)	28.13 (4.85)	29.16 (5.17)
Total physical activity per week, MET hour	47.61 (46.37)	45.57 (45.63)	43.48 (44.95)	40.92 (44.36)
Waist circumstance, cm	85.29 (11.77)	88.84 (12.89)	92.22 (13.39)	94.92 (13.62)
Socioeconomic status				
Townsend deprivation index	−1.43 (3.00)	−1.43 (3.01)	−1.32 (3.08)	−1.07 (3.22)
Most deprived fifth, *n* (%)	21,579 (20.63)	21,912 (20.78)	21,047 (19.95)	19,622 (18.60)
Professional qualification, college/university degree, *n* (%)	41,047 (39.24)	35,970 (34.11)	32,362 (30.67)	28,846 (27.35)
Smoking, *n* (%)				
Never	63,592 (60.80)	59,606 (56.53)	56,241 (53.30)	52,756 (50.02)
Previous	32,052 (30.64)	35,276 (33.46)	37,550 (35.59)	39,577 (37.52)
Current	8,952 (8.56)	10,556 (10.01)	11,729 (11.12)	13,145 (12.46)
Alcohol intake, *n* (%)				
Never	4,875 (4.66)	4,678 (4.44)	4,531 (4.29)	4,382 (4.15)
Previous	4,268 (4.08)	3,518 (3.34)	3,437 (3.26)	3,600 (3.41)
Current	95,453 (91.26)	97,242 (92.22)	97,552 (92.45)	97,496 (92.43)
Glycated hemoglobin, mmol/mol	34.69 (5.33)	35.57 (5.76)	36.44 (6.76)	37.54 (8.37)
C‐reactive protein, mg/L	1.67 (3.31)	2.17 (3.74)	2.74 (4.21)	3.60 (5.24)
Total bilirubin, umol/L	9.30 (4.61)	9.21 (4.41)	9.08 (4.35)	9.07 (4.38)
Alkaline phosphatase, U/L	75.86 (21.04)	80.33 (21.44)	83.86 (22.58)	92.73 (31.93)
Aspartate aminotransferase, U/L	23.23 (6.36)	24.44 (6.71)	25.94 (7.30)	31.15 (15.25)
Alanine aminotransferase, U/L	17.23 (6.72)	20.27 (8.58)	24.05 (11.21)	32.88 (20.44)
Triglycerides, mmol/L	1.37 (0.72)	1.62 (0.88)	1.86 (1.02)	2.11 (1.22)
Cholesterol, mmol/L	5.45 (1.04)	5.67 (1.10)	5.76 (1.15)	5.87 (1.22)
Low‐density lipoprotein direct, mmol/L	3.38 (0.79)	3.55 (0.84)	3.62 (0.88)	3.67 (0.92)

*Note*: Mean (standard deviation). The quartile of GGT level across genders were calculated separately. In women: Q1 (<16 U/L), Q2 (16–21.3 U/L), Q3 (21.3–31.6 U/L), and Q4 (≥31.6 U/L). In men: Q1 (<23.7 U/L), Q2 (23.7–33.1 U/L), Q3 (33.1–50.2 U/L), and Q4 (≥50.2 U/L).

Abbreviations: MET hour, metabolic equivalents hour.

### 3.2 Association between serum gamma‐glutamyl transpeptidase level and incident pancreatic cancer

Over a median follow‐up time of 7.16 years, a total of 586 incident pancreatic cancer cases were identified. Among them, 93 (0.09%) were in the bottom GGT quartile group and 184 (0.17%) in the top GGT quartile group (Table 1). When considering GGT level as a continuous variable, adjusting for gender, age at recruitment, UK Biobank Assessment Centre, Townsend deprivation index at recruitment and qualifications, BMI, waist circumference, physical activity, smoking status, alcohol drinking status, and glycated hemoglobin hba1c, a SD increment of log2 GGT was associated with a higher risk of pancreatic cancer (model 2, HR = 1.13, 95%CI: 1.03–1.25) (Table 2). The positive association remained unchanged when additional adjustment was made for markers of inflammation and that possibly interrelate/cross talk with GGT pathway including C‐reactive protein, total bilirubin, alkaline phosphatase, alanine aminotransferase, aspartate aminotransferase, cholesterol, triglycerides, and low‐density lipoprotein direct in the fully adjusted Cox model (model 3, HR = 1.14, 95%CI: 1.02–1.28). The identical associations were observed for men (model 3, HR = 1.16, 95%CI: 1.00–1.34) and women (model 3, HR = 1.13, 95%CI: 0.97–1.33). When GGT level was analyzed as a categorical variable, participants in the top quartile of GGT level had an increased risk of pancreatic cancer by 68% compared with those in the bottom quartile. (model 3, HR = 1.68, 95%CI: 1.22–2.30, *p*‐trend = 0.003). The similar associations were observed for men (model 3, HR = 1.72, 95%CI: 1.14–2.61, *p*‐trend = 0.016) and women (model 3, HR = 1.75, 95%CI: 1.06–2.88, *p*‐trend = 0.026).

**TABLE 2:** Associations between serum gamma‐glutamyl transpeptidase level and risk of pancreatic cancer in the UK Biobank

Quartile cutpoints, U/L	Q1	Q2	Q3	Q4	*P* ‐trend^d^	HR per 1‐SD increment GGT (Log2 transformation)	HR per 1‐SD Increment GGT (Log2 transformation) *p* value
Women	<16	<21.3	<31.6	≥31.6			
Men	<23.7	<33.1	<50.2	≥50.2			
Total							
Crude model	1 (ref)	1.53 (1.18–1.99)	1.77 (1.38–2.29)	1.97 (1.54–2.53)	<0.001	1.25 (1.16–1.34)	<0.001
		0.001	<0.001	<0.001			
Model 1^a^	1 (ref)	1.37 (1.05–1.78)	1.54 (1.19–1.98)	1.71 (1.33–2.20)	0.002	1.17 (1.08–1.26)	<0.001
		0.019	0.001	<0.001			
Model 2^b^	1 (ref)	1.27 (0.94–1.71)	1.38 (1.02–1.85)	1.64 (1.23–2.20)	0.003	1.13 (1.03–1.25)	0.011
		0.122	0.035	0.001			
Model 3^c^	1 (ref)	1.28 (0.95–1.73)	1.39 (1.02–1.88)	1.68 (1.22–2.30)	0.003	1.14 (1.02–1.28)	0.025
		0.110	0.035	0.001			
Women							
Crude model	1 (ref)	1.93 (1.27–2.94)	2.26 (1.50–3.41)	2.75 (1.85–4.10)	<0.001	1.28 (1.16–1.42)	<0.001
		0.002	<0.001	<0.001			
Model 1^a^	1 (ref)	1.59 (1.04–2.42)	1.73 (1.14–2.61)	2.02 (1.35–3.02)	0.003	1.19 (1.07–1.33)	0.002
		0.031	0.010	0.001			
Model 2^b^	1 (ref)	1.27 (0.94–1.71)	1.38 (1.02–1.85)	1.64 (1.23–2.20)	0.009	1.16 (1.02–1.33)	0.027
		0.122	0.035	0.001			
Model 3^c^	1 (ref)	1.32 (0.81–2.15)	1.25 (0.76–2.06)	1.75 (1.06–2.88)	0.026	1.13 (0.97–1.33)	0.125
		0.271	0.370	0.027			
Men							
Crude model	1 (ref)	1.31 (0.94–1.84)	1.51 (1.09–2.09)	1.55 (1.12–2.14)	0.020	1.12 (1.01–1.25)	0.026
		0.110	0.013	0.008			
Model 1^a^	1 (ref)	1.25 (0.89–1.75)	1.45 (1.05–2.01)	1.56 (1.13–2.16)	0.011	1.13 (1.02–1.26)	0.020
		0.191	0.025	0.008			
Model 2^b^	1 (ref)	1.25 (0.85–1.84)	1.48 (1.02–2.15)	1.56 (1.07–2.28)	0.034	1.10 (0.98–1.24)	0.117
		0.251	0.041	0.022			
Model 3^c^	1 (ref)	1.28 (0.87–1.88)	1.54 (1.05–2.26)	1.72 (1.14–2.61)	0.016	1.16 (1.00–1.34)	0.051
		0.209	0.028	0.010			

*Note*: Crude model: None.

Abbreviations: HR, hazard ratio; SD, standard deviation.

^a^ Model 1: Adjusted for gender, age at recruitment (continuous variable, years), UK Biobank Assessment Centre (England, Scotland, Wales), Townsend deprivation index at recruitment (continuous variable), qualifications (college, others, unknown).

^b^ Model 2: model 1 + body mass index (continuous variable, kg/m2), waist circumference (continuous variable, cm), total physical activity per week (continuous variable, MET hours), smoking status (never, former, current), alcohol drinking status (never, former, current), levels of glycated hemoglobin hba1c (continuous variable, mmol/mol).

^c^ Model 3: model 2 + C‐reactive protein (continuous variable, mg/L), total bilirubin (continuous variable, umol/L), alkaline phosphatase (continuous variable, U/L), alanine aminotransferase (continuous variable, U/L), aspartate aminotransferase (continuous variable, U/L), cholesterol (continuous variable, mmol/L), triglycerides (continuous variable, mmol/L), low‐density lipoprotein direct (continuous variable, mmol/L).

^d^ Test for trend based on variable containing median value for each quartile.

**FIGURE 2:**
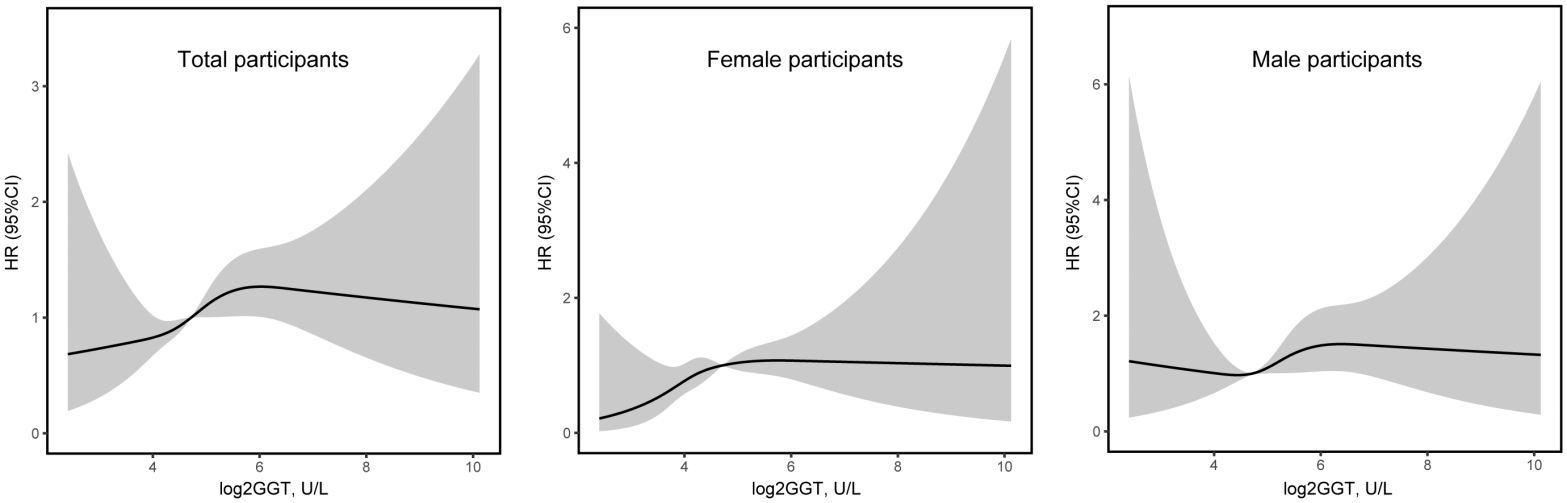
Associations of log2 transformation of serum gamma‐glutamyl transpeptidase level with risk of pancreatic cancer. Cubic spline models were fully adjusted for gender, age (continuous variable, years), UK Biobank assessment centre (England, Scotland, Wales), Townsend deprivation index at recruitment (continuous variable), qualifications (college, others, unknown), body mass index (continuous variable, kg/m^2^), waist circumference (continuous variable, cm), total physical activity (continuous variable, MET hours), smoking status (never, former, current), alcohol drinking (never, former, current), glycated hemoglobin hba1c (continuous variable, mmol/mol), C‐reative protein (continuous variable, mg/L), total bilirubin (continuous variable, μmol/L), alkaline phosphatase (continuous variable, U/L), alanine aminotrnasferase (continuous variable, U/L), aspartate aminotrnasferase (continuous variable, U/L), cholesterol (continuous variable, mmol/L), triglycerides (continuous variable, mmol/L), LDL direct (continuous variable, mmol/L). Non‐linear *p*‐values were 0.249, 0.282, 0.438 for GGT in the total population, female participants, and male participants, respectively. Abbreviations: CI, confidence interval; HR, hazard ratio.

Associations of log2 transformation of serum GGT level with incident pancreatic cancer appeared to be generally linear (Figure 2).

### 3.3 Subgroup analysis

Stratification analysis revealed a notable association between serum GGT and pancreatic cancer risk in the population aged ≥60 years (Q4 vs. Q1, HR = 1.94, 95%CI: 1.34–2.81), never/current smokers (Q4 vs. Q1, HR = 2.33, 95%CI: 1.44–3.76; Q4 vs. Q1, HR = 2.77, 95%CI: 1.25–6.12), current alcohol drinkers (Q4 vs. Q1, HR = 1.72, 95%CI: 1.24–2.40), those with low BMI <25 kg/m^2^ (Q4 vs. Q1, HR = 2.22, 95%CI: 1.17–4.22), and without new‐onset diabetes within 3 years before the cancer diagnosis (Q4 vs. Q1, HR = 1.65, 95%CI: 1.20–2.27). GGT's impact on pancreatic carcinogenesis was not influenced by age, smoking status (never, ever, and current), alcohol consumption status (never, ever, and current), or BMI in either subgroup analysis (all *p* for interactions >0.05) (Table 3).

**TABLE 3:** Subgroup analysis

Subgroup analysis	Number of pancreatic cancer cases/Person‐years	Q1	Q2	Q3	Q4	****P**** for trend^a^	HR per 1‐SD Increment GGT (Log2 transformation)	HR per 1‐SD Increment GGT (Log2 transformation) *p* value	*P* for interaction
Age at recruitment, years									
<60	184/171,1481	1	1.23 (0.71–2.16)	1.49 (0.86–2.57)	1.33 (0.73–2.42)	0.64	1.14 (0.92–1.40)	0.24	0.30
≥60	402/1,179,023	1	1.51 (1.06–2.14)	1.44 (1.00–2.06)	1.94 (1.34–2.81)	0.002	1.16 (1.02–1.32)	0.03	
Smoking status									
Never	262/1,602,418	1	1.43 (0.89–2.29)	1.49 (0.93–2.40)	2.33 (1.44–3.76)	<0.001	1.24 (1.05–1.46)	0.01	0.16
Former	226/985,421	1	1.18 (0.75–1.85)	1.05 (0.66–1.68)	0.99 (0.60–1.64)	0.71	0.99 (0.81–1.20)	0.90	
Current	98/302,664	1	1.81 (0.86–3.80)	2.10 (0.99–4.46)	2.77 (1.25–6.12)	0.02	1.20 (0.90–1.60)	0.21	
Alcohol consumption									
Never	22/127,474	1	1.45 (0.37–5.71)	1.50 (0.35–6.52)	0 (9.732107 e‐19‐4.820170 e+11)	0.09	0.54 (0.24–1.23)	0.14	0.08
Former	29/100,668	1	0.63 (0.14–2.89)	1.47 (0.40–5.42)	2.47 (0.63–9.59)	0.07	1.30 (0.76–2.22)	0.33	
Current	535/2,662,361	1	1.36 (0.99–1.86)	1.33 (0.96–1.83)	1.72 (1.24–2.40)	0.004	1.20 (0.90–1.60)	0.21	
Body mass index, kg/m^2^									
<25	153/955,735	1	1.65 (0.86–3.15)	2.03 (1.09–3.80)	2.22 (1.17–4.22)	0.04	1.19 (0.97–1.46)	0.10	0.15
≥25	433/1,934,768	1	1.19 (0.85–1.68)	1.46 (1.04–2.04)	1.40 (0.97–2.01)	0.13	1.11 (0.98–1.27)	0.11	
New‐onset diabetes before the cancer diagnosis within 3 years									
Positive	6/139	1	0.00 (0.00‐Inf)^b^	0.00 (0.00‐Inf)	0.00 (0.00‐Inf)	—	—	—	—
Negative	580/2,890,365	1	1.28 (0.94–1.73)	1.33 (0.98–1.81)	1.65 (1.20–2.27)	0.004	1.14 (1.01–1.27)	0.03	

*Note*: Adjusted for gender, age at recruitment (continuous variable, years), UK Biobank Assessment Centre (England, Scotland, Wales), Townsend deprivation index at recruitment (continuous variable), qualifications (college, others, unknown), body mass index (continuous variable, kg/m^2^), waist circumference (continuous variable, cm), total physical activity per week (continuous variable, MET hours), smoking status (never, former, current), alcohol drinking status (never, former, current), levels of glycated hemoglobin hba1c (continuous variable, mmol/mol), C‐reactive protein (continuous variable, mg/L), total bilirubin (continuous variable, umol/L), alkaline phosphatase (continuous variable, U/L), alanine aminotransferase (continuous variable, U/L), aspartate aminotransferase (continuous variable, U/L), cholesterol (continuous variable, mmol/L), triglycerides (continuous variable, mmol/L), low‐density lipoprotein direct (continuous variable, mmol/L).

Abbreviations: HR, hazard ratio; SD, standard deviation.

^a^ Test for trend based on variable containing median value for each quartile.

^b^ The margin of error (95%CI: 0.00‐Inf) is large here primarily due to the small sample size.

### 3.4 Sensitivity analysis

After considering  1‐year lag period, we still detected a positive correlation between serum GGT level and pancreatic cancer risk (Q4 vs. Q1: HR 1.66 95%CI 1.20–2.29). When removing participants with new‐onset diabetes before the cancer diagnosis within 3 years, or participants with IBD, or participants with liver or bile duct‐related disease and liver‐toxicity medication use, the results remained essentially unchanged. (Q4 vs. Q1: HR 1.65 95%CI 1.20–2.27; Q4 vs. Q1: HR 1.69 95%CI 1.23–2.32; Q4 vs. Q1: HR 2.15 95%CI 1.30–3.55) After additionally adjusting dietary lifestyle factors or first‐degree family history of digestive cancer, the impact of serum GGT on incident pancreatic cancer held solid. (Q4 vs. Q1: HR 1.65 95%CI 1.19–2.29; Q4 vs. Q1: HR 1.67 95%CI 1.22–2.29) The identical associations were found in gender stratification. (Table 4).

**TABLE 4:** Sensitivity analysis

Gamma‐glutamyl transpeptidase concentration, U/L	Total		Men		Women	
	Number of cases/Person‐years	HR (95% CI)	Number of cases/Person‐years	HR (95% CI)	Number of cases/Person‐years	HR (95% CI)
Limiting the participants in individuals with follow‐up above 1 year^a^						
Quartile 1	90/616,707	1	58/287,158	1	32/329,549	1
Quartile 2	140/618,826	1.28 (0.94–1.74)	77/287,550	1.27 (0.86–1.88)	63/331,276	1.34 (0.81–2.21)
Quartile 3	157/618,832	1.34 (0.98–1.82)	84/288,056	1.47 (0.99–2.17)	73/330,776	1.25 (0.75–2.07)
Quartile 4	177/616,114	1.66 (1.20–2.29)	87/286,895	1.67 (1.10–2.56)	90/329,219	1.79 (1.08–2.96)
Limiting the participants in individuals without new‐onset diabetes at least 3 years before the cancer diagnosis^b^						
Quartile 1	93/721,107	1	60/336,102	1	33/385,005	1
Quartile 2	142/723,999	1.28 (0.94–1.73)	78/336,667	1.28 (0.87–1.88)	64/387,332	1.31 (0.80–2.14)
Quartile 3	162/724,043	1.33 (0.98–1.81)	89/337,239	1.49 (1.02–2.20)	73/386,805	1.19 (0.72–1.96)
Quartile 4	183/721,216	1.65 (1.20–2.27)	92/336,073	1.70 (1.12–2.57)	91/385,143	1.73 (1.05–2.84)
Limiting the participants in in individuals without IBD^c^						
Quartile 1	91/713,055	1	60/332,030	1	31/381,025	1
Quartile 2	141/716,366	1.27 (0.94–1.73)	77/332,690	1.24 (0.84–1.82)	64/383,676	1.37 (0.84–2.26)
Quartile 3	164/713,502	1.39 (1.02–1.88)	91/333,106	1.53 (1.04–2.25)	73/380,396	1.26 (0.76–2.09)
Quartile 4	182/713,739	1.69 (1.23–2.32)	92/331,368	1.69 (1.12–2.56)	90/382,371	1.82 (1.10–3.02)
Limiting the participants without liver or bile duct disease or liver‐toxicity medication use^d^						
Quartile 1	35/551,605	1	22/250,395	1	13/301,210	1
Quartile 2	61/559,924	1.56 (0.96–2.53)	28/254,553	1.51 (0.79–2.89)	33/305,371	1.63 (0.79–3.39)
Quartile 3	72/554,240	1.73 (1.07–2.80)	42/253,015	2.06 (1.10–3.87)	30/301,225	1.37 (0.64–2.92)
Quartile 4	84/555,122	2.15 (1.30–3.55)	43/252,953	2.27 (1.16–4.44)	41/302,169	2.17 (1.03–4.60)
Additionally adjusting dietary lifestyle factors^e^						
Quartile 1	93/721,110	1	60/336,104	1	33/385,006	1
Quartile 2	143/724,019	1.31 (0.96–1.79)	79/336,682	1.26 (0.85–1.89)	64/387,337	1.43 (0.86–2.37)
Quartile 3	166/724,086	1.34 (0.97–1.83)	91/337,265	1.43 (0.96–2.14)	75/386,821	1.28 (0.76–2.14)
Quartile 4	184/721,289	1.65 (1.19–2.29)	93/336,111	1.63 (1.06–2.50)	91/385,178	1.80 (1.07–3.02)
Additionally adjusting first‐degree family history of digestive cancer						
Quartile 1	93/721,110	1	60/336,104	1	33/385,006	1
Quartile 2	143/724,019	1.28 (0.94–1.73)	79/336,682	1.28 (0.87–1.88)	64/387,337	1.31 (0.80–2.15)
Quartile 3	166/724,086	1.38 (1.02–1.87)	91/337,265	1.54 (1.05–2.26)	75/386,821	1.25 (0.76–2.05)
Quartile 4	184/721,289	1.67 (1.22–2.29)	93/336,111	1.72 (1.14–2.60)	91/385,178	1.74 (1.06–2.86)

*Note*: Adjusted for gender, age (continuous variable, years), UK Biobank assessment centre (England, Scotland, Wales), Townsend deprivation index at recruitment (continuous variable), qualifications (college, others, unknown), body mass index (continuous variable, kg/m^2^), waist circumference (continuous variable, cm), physical activity (continuous variable, MET hours), smoking status (never, former, current), alcohol drinking (never, former, current), glycated hemoglobin hba1c (continuous variable, mmol/mol), C‐reative protein (continuous variable, mg/L), total bilirubin (continuous variable, μmol/L), alkaline phosphatase (continuous variable, U/L), alanine aminotrnasferase (continuous variable, U/L), aspartate aminotrnasferase (continuous variable, U/L), cholesterol (continuous variable, mmol/L), triglycerides (continuous variable, mmol/L), LDL direct (continuous variable, mmol/L).

Abbreviations: CI, confidence interval; HR, hazard ratio.

^a^ After excluding participants with less than 1 year of follow‐up, 418,782 individuals remained in the analysis.

^b^ After excluding participants with new‐onset diabetes within 3 years before the cancer diagnosis, 420,929 individuals remained in the analysis.

^c^ After excluding participants with IBD, 416,005 individuals remained in the analysis.

^d^ After excluding participants with liver or bile duct disease (ICD 10 code: K70‐K77, K80‐K83) or liver‐toxicity medication use (paracetamol, aspirin, ibuprofen, simvastatin, carbamazepine), 322,788 individuals remained in the analysis.

^e^ Additionally adjust dietary lifestyle factors, including raw and cooked vegetable intake, fresh and dried fruit intake, frequency of poultry consumption.

## 4 DISCUSSION

In this large prospective study based on the UK Biobank data, our findings indicated that elevated serum GGT level predicted a higher risk of pancreatic cancer after fully adjusting for multiple confoundings. When sensitivity analyses were applied, the results remained robust, supporting a positive association between circulating GGT level and pancreatic cancer occurrence.

The studied association between GGT level and pancreatic cancer incidence in other population‐based researches have failed to reach consistent conclusions. In the Swedish AMORIS study with 545,460 persons, the HR for developing pancreatic cancer per log unit increase in GGT level was 1.36 (95%CI: 1.22–1.52).^11^ In the Ohsaki Cohort with 15,031 Japanese adults, there was a 89% higher risk of pancreatic cancer among those in the highest GGT quartile as compared to those in the lowest quartile, but with null hypothesis (HR =1.89, 95%CI: 0.81–4.38).^10^ According to this large scale UK Biobank cohort with 421,032 participants, our finding supported the evidence of raised pancreatic cancer risk at elevated GGT level among European population.

Xiao et al. reported that metastatic pancreatic cancer patients with higher GGT level had poorer overall survival outcomes. After controlling for possible confounding variables, serum GGT > 48 U/L led to a HR of 1.53 (95%CI: 1.19–1.97) for mortality risk. However, some potential confounders were unavailable with no adjustments made for smoking history, alcohol consumption, or tumor location, etc.^8^

To the best of our knowledge, few studies have examined gender disparities in serum GGT levels on the risk of pancreatic cancer. In this study, it was not found that GGT level was correlated with pancreatic cancer incidence in a sex‐aligned fashion. The observed multivariate HR was 1.72 (Q4 vs. Q1, 95%CI: 1.14–2.61) in men and 1.75 (Q4 vs. Q1, 95%CI: 1.06–2.88) in women. In Japanese population, when stratified the gender, the multivariate HR was 1.16 (95%CI: 0.90–1.50) in men and 1.22 (95%CI: 0.92–1.62) in women, supporting the notion that GGT level and cancer incidence are not sex‐dependent.^10^ However, a population‐based study in Korea found that the association between GGT and digestive cancer incidence was dominant in men than that in women.^20^ In Swedish population, the HRs of overall cancer risks for highly elevated level of GGT (>72 U/L) versus normal level (<18 U/L) were 1.49 (95%CI: 1.17–1.89) for men and 1.14 (95%CI: 0.87–1.50) for women.^11^ However, in the latter study, they did not fully consider the sex disparities in serum GGT level, ignoring that men have higher GGT activity than women.

The subgroup analysis found an association between serum GGT and pancreatic cancer incidence in the population aged ≥60 years or never/current smokers, current alcohol drinkers, with low BMI (<25 kg/m^2^) and without new‐onset diabetes before the cancer diagnosis within 3 years, although other subgroup analyses with smaller sample size could not show sufficient evidence. There is no interaction among the subgroups including age, smoking status, alcohol drinking status, or BMI. Similarly, in National Health Insurance Service (NHIS) with Korean Cancer Prevention Study (KCPS), associations between serum GGT and site‐specific cancers were diminished in the high BMI (≥25 kg/m^2^) and the younger group.^9^ In accord with study by Lee et al,^20^ the prominent effect of GGT on pancreatic cancer initiation was not seen in people who had never smoked or drunk, but in current drinkers. Nevertheless, Mok et al. found that baseline GGT and cancer risk were associated irrespective of drinking and smoking habits.^9^ Whether alcohol intake acts as a mediator in the association between serum GGT level and risk of pancreatic cancer is unknown and ambiguous.^20^

Potential biological mechanisms have been disclosed to understand the serum GGT's role in pancreatic cancer initiation. In the presence of elevated GGT levels, red blood cell membranes could be damaged and the released hazardous transition metals potentially activates chain pro‐oxidant reactions. When levels of oxidative stress increase, reactive oxygen species (ROS) are formed,^7^ which could cause genetic instability and drive precancerous lesions.^21^ However, the above hypothesis supported elevated GGT is linked to cancer of any kind. Previous studies found GGT was only positively associated with alcohol‐related cancers such as pancreatic cancer, etc.^10^ The etiological role of serum GGT through underlying ROS biology inducing pancreatic carcinogenesis is necessary to be recognized, requiring future in‐depth experimental investigation.

Since in clinical practice, serum GGT measurements are commonly carried out as a quick and inexpensive routine test, when individuals detected with high GGT level, timely intervention could potentially prevent the pancreatic cancer onset. It is worth noting that, the beneficial effect of adhering to a healthy lifestyle such as the consumption of fruit, vegetables,^22^ supplementation with antioxidants like vitamin C,^23^ Q10H2,^24^ treatment with GGT inhibitor,^25^ etc. contributed to lowering GGT level. Still, these modifiable strategies should be validated their rationale and net benefits with caution before they are recommended in routine health care settings.

A strength of our study is relied on a nationwide prospective cohort, which gathered extensive data on a broad spectrum of covariates. In this way, we maintained adequate control of potential confounding factors for the associations of interest. Additionally, we provided comprehensive follow‐ups and robust sensitivity analyses. Some limitations of the study should also be noted. No repeated measurements for GGT were available. Besides, pancreatic cancer is relatively rare in Europe. This study was dependent on genetic data of European descent, so generalizability of our findings to other ethnicity is a concern. Further studies are required to investigate whether it hold valid in population with different genetic backgrounds. Finally, other cohorts will be expected to be applied to verify our results and provide more confidence.

## 5 CONCLUSION

Serum GGT level was a positive indicator of pancreatic cancer occurrence. Individuals with high risk of pancreatic cancer might benefit from close surveillance with serum GGT measurements. Further studies need to explore the potential causality of serum GGT level on incident pancreatic cancer.

## AUTHOR CONTRIBUTIONS

**Weiting Liao:** Conceptualization (lead); investigation (lead); methodology (lead); visualization (lead); writing – original draft (lead). **Yu Yang:** Conceptualization (equal); writing – original draft (equal); writing – review and editing (equal). **Huazhen Yang:** Data curation (equal); investigation (equal); resources (equal). **Yuanyuan Qu:** Project administration (equal); resources (equal); writing – review and editing (equal). **Huan Song:** Conceptualization (lead); methodology (lead); resources (lead); supervision (lead); visualization (lead); writing – review and editing (lead). **Qiu Li:** Conceptualization (lead); funding acquisition (lead); project administration (lead); resources (lead); supervision (lead); validation (lead); writing – review and editing (lead).

## FUNDING INFORMATION

The work was financially supported by National Science and Technology Major Projects of China (No. 2017ZX09304023), the 1.3.5 project for disciplines of excellence, West China Hospital, Sichuan University (No. ZYJC18010). The funding had no role in study design, data collection, analysis or interpretation, or in writing the report.

## CONFLICT OF INTEREST

The authors declare no potential conflicts of interest.

## ETHICAL APPROVAL STATEMENT

The UK Biobank obtained written informed consent from each participant, and the study has been ethically approved by the NHS National Research Ethics Service (16/NW/0274). The biomedical research ethics committee of West China Hospital has also approved this study (2019–1171).

## Data Availability

UK Biobank is an open‐access resource. Bona fide researchers can apply to use the UK Biobank dataset by registering and applying at http://ukbiobank.ac.uk/register‐apply. The data that support the findings in this study are available from the corresponding author upon reasonable request.
